# Rate of Congenital Toxoplasmosis in Large Integrated Health Care Setting, California, USA, 1998–2012

**DOI:** 10.3201/eid2009.131919

**Published:** 2014-09

**Authors:** Jeffrey L. Jones, Valentina A. Shvachko, E. Elizabeth Wilkins, Randy Bergen, M. Michele Manos

**Affiliations:** Centers for Disease Control and Prevention, Atlanta, Georgia, USA (J.L. Jones, E.E. Wilkins);; Kaiser Permanente Division of Research, Oakland, California, USA (V.A. Shvachko ,R. Bergen, M.M. Manos)

**Keywords:** Toxoplasmosis, *Toxoplasma gondii*, parasite, congenital, California

## Abstract

Rate of Congenital Toxoplasmosis, California

**To the Editor:** Although congenital toxoplasmosis occurs throughout the United States, little information is available about the rates of diagnosed illness in most of the nation, including California. Infection usually occurs by ingestion of undercooked meat and unwashed fruits and vegetables or exposure to soil or water contaminated with cat feces. Congenital transmission can occur when a woman is infected with *Toxoplasma gondii* during, or just before, pregnancy. Approximately 91% of women of childbearing age in the United States are susceptible to *T. gondii* infection (*1*). The United States has a low prevalence of *T. gondii* infection compared with many areas of the world (*2*). Severe congenital toxoplasmosis can result in hydrocephalus, retinochoroiditis that affects vision, microcephalus, seizures, hepatosplenomegaly, icterus, psychomotor retardation, and other sequelae (*3*). Infants with congenital toxoplasmosis are most often asymptomatic at birth; however, when severe symptoms occur, they are usually recognized and the condition diagnosed by the time the child is 2 years of age (*3*).

Our goal was to determine the rate of clinically identified cases of congenital toxoplasmosis in children from birth to 2 years of age within the Northern California Kaiser Permanente Medical Care Program (KPNC) during a 15-year period. KPNC is a group health plan that provides care for >3.2 million residents of northern California. The KPNC membership represents ≈30% of the insured population in the region and is demographically similar to the residents of the counties served except that the very poor and very wealthy are underrepresented (*4*).

We studied live births and infants during 1998–2012, the most recent 15-year period for which records were available and considered complete. We identified potential cases from KPNC electronic medical record databases and confirmed them by reviewing electronic and paper records. The system documents outside services, identified by the corresponding diagnostic codes or laboratory test codes. Eligible case-patients were infants, defined as <24 months of age, at the time of meeting any potential case criterion. We identified all births in which ICD-9-CM diagnostic codes for the mother or the infant included the following: 130-130.9 (toxoplasmosis), 771.2a (a special KPNC subset code specifying toxoplasmosis), and those with the more general 771.2 (congenital infections specific to the perinatal period) code; for the latter, an external special test for toxoplasmosis was assessed). We also identified all infants for whom any toxoplasmosis laboratory test had been ordered that had >1 of 23 specific KPNC laboratory codes for related serologic and PCR tests. We considered clinically confirmed case-patients to be infants with positive *T. gondii* IgM and/or IgA tests at <6 months of age, persistent IgG at >12 months of age, PCR–positive results for *T. gondii*, or diagnosis and care of toxoplasmosis-related conditions. To calculate 95% confidence intervals for rates, we used the exact binomial method.

During the 15-year study period, there were 521,655 live births at KPNC facilities and 2,010 infants received >1 test for toxoplasmosis. Ten infants met the potential case criteria of diagnostic codes; no additional patients met any of the case criteria by age 2 years. After electronic and paper charts were reviewed, 2 cases of congenital toxoplasmosis were confirmed. One case was diagnosed in 2003, the other in 2011. Both case-patients were girls: 1 was of Hispanic ethnicity and the other was of mixed Filipino-White heritage; both had IgG persistently detected beyond 12 months of age and were monitored clinically for retinochoroiditis. Their charts contained no information regarding maternal exposure or risk factors. During the 15-year period, the rate of diagnosed congenital toxoplasmosis was 3.8 (95% CI 1.5–9.2) per million live births. There were no infant deaths for which congenital toxoplasmosis was recorded as a cause. We were unable to study fetal deaths because the corresponding cause-of-death codes were not readily available.

Historically, the lowest prevalence of *T. gondii* infection has been recorded in the western United States (*5*). The rate of clinically apparent congenital toxoplasmosis in this study was lower than that found during the late 1980s through early 1990s in the New England Newborn Screening Program initially after birth (2 per 521,555 live births [3.8 per million] versus 5 per 635,000 live births [7.9 per million], respectively) (*6*). However, the prevalence of *T. gondii* infection has decreased in the United States since the 1990s (*1*).

Our study is subject to several limitations. Our approach would only detect clinically apparent cases, and the results should be considered a minimal estimate of congenital infection. Some cases may not have been recorded in the electronic system, but this omission is not likely for severe illness, repeated hospital or clinic visits, or outside consultation. The small number of cases makes the rate of diagnosed congenital toxoplasmosis somewhat imprecise; a few missed cases would increase the rate considerably. In addition, we were not able to evaluate fetal deaths; however, stillbirth is reportedly a rare complication of congenital toxoplasmosis (*7*). Although we found a low rate of diagnosed congenital toxoplasmosis in northern California, population-based studies to evaluate rates of the disease in other geographic areas would be beneficial.
